# Complete Genome Sequence of *Rhodopseudomonas* sp. Strain P2A-2r, Isolated from Arctic Soil

**DOI:** 10.1128/mra.00013-23

**Published:** 2023-02-22

**Authors:** Polina Guro, Ekaterina Karaevskaya, Denis Karlov, Irina Kuznetsova, Anna Sazanova, Vera Safronova

**Affiliations:** a All-Russia Research Institute for Agricultural Microbiology (ARRIAM), St. Petersburg, Russian Federation; b Arctic and Antarctic Research Institute, St. Petersburg, Russian Federation; Wellesley College

## Abstract

Microorganisms of extremely cold habitats are unique objects for studying their biogeochemical properties and mechanisms. Here, we present the complete genome sequence of the strain *Rhodopseudomonas* sp. P2A-2r, isolated from arctic soil in Svalbard, Norway. The genome consists of a 6.7-Mbp circular chromosome.

## ANNOUNCEMENT

The permafrost of the Svalbard Archipelago is the warmest one in the Arctic compared with those of other areas at the same high latitude. Due to the influence of sea currents and air masses moving from the Atlantic with the West Svalbard current, a unique arctic ecological niche is formed (1). The soil sample has been collected from a depth of 10 cm (78.02319° N, 14.30067° E, 2.0 MASL) near the Grøn River Mouth (southern shore of Isfjorden Bay, Norway) and was a wet loam with root fragments. A soil suspension (0.11 g of soil sample per 5 mL sterile 0.9% NaCl solution) was prepared, and 150 μl of it was then plated in R2A medium (Difco, USA) and incubated at 5°C. Individual observed colonies were transferred to a new plate to obtain a pure culture. Genomic DNA was isolated using the DNeasy blood and tissue kit (Qiagen, Germany) according to the manufacturer's recommendations without size selection from a pure culture grown in liquid R2A medium at 28°C for 48 h. The SQK-LSK109 ligation sequencing kit (Oxford Nanopore Technologies [ONT], UK) with the EXP-NBD104 native barcoding expansion 1–12 kit (ONT) was used to prepare the library according to the manufacturer’s instructions, skipping the DNA-shearing step. The long-read whole genome was sequenced using a MinION sequencer with flow cell R.9.4.1 (ONT). The read base calling, demultiplexing, and adapter trimming were performed using the Guppy program (v 5.0.11) (2). Quality control of the raw reads was provided by NanoStat (v 1.6.0) (3). The sequencing produced 48,183 reads with an average read length of 11,569 bp and an average quality of 11.4. The genome sequence was obtained by the Flye (v 2.9) assembler and produced 1 contig (4). The circular chromosome was generated by Circlator (v 1.5.5) and was rotated with the *dnaA* gene as the starting position (5). Default parameters were used for all software unless otherwise noted. Genomic statistics for assembly were measured using QUAST (v 5.0.2) (6). The total size of the assembly and *N*_50_ value is 6,737,367 bp with an average coverage of 80× and a GC content of 63.87%. The assembly was annotated using the NCBI PGAP (v 6.3) software (7). Of the 6,468 predicted total genes, 5,786 are protein-coding genes and 65 are RNA genes (49 tRNAs, 12 rRNAs, and 4 noncoding RNAs [ncRNAs]).

Strain P2A-2r was identified as a *Rhodopseudomonas* sp. by 16S rRNA BLASTn comparison and average nucleotide identity (ANI) analysis in the JSpeciesWS online server (8, 9). The 16S rRNA gene sequence of P2A-2r was closely related (98.25% identity) to the type strain Rhodopseudomonas pseudopalustris DSM 123 (GenBank accession number NR_122099.1). ANI values with the closest type strain DSM 123 showed 76.52% ANI based on BLAST (ANIb) identity and 84.41% ANI based on MUMmer (ANIm) identity. Among the annotated genes, key nitrogen fixation genes (*fixABCX*, *fixLJ*, *fixNOQPGHIS*, *nifAHENX*, *nifUSTBZ*, and *nifOHQW*) were identified, as well as some nodulation genes (*nodBCD*, *nodPQ*, and *nodVW*) and a multiple putative genes of metal resistance and stress response.

### Data availability.

All data are available at GenBank under BioProject accession number PRJNA881974. The BioSample accession number is SAMN31488911. The raw MinION data can be found under number SRR22403519 and the assembly accession number is CP110389.1.

## References

[1] Forman SL, Lubinski DJ, Ingolfsson O, Zeeberg JJ, Snyder JA, Siegert MJ, Matishov GG. 2004. A review of postglacial emergence on Svalbard, Franz Josef Land and Novaya Zemlya, northern Eurasia. Quat Sci Rev 23:1391–1434. doi:10.1016/j.quascirev.2003.12.007.

[2] Wick RR, Judd LM, Holt KE. 2019. Performance of neural network basecalling tools for Oxford Nanopore sequencing. Genome Biol 20:129. doi:10.1186/s13059-019-1727-y. 31234903 PMC6591954

[3] Coster WD, D’Hert S, Schultz DT, Cruts M, Broeckhoven CV. 2018. NanoPack: visualizing and processing long-read sequencing data. Bioinformatics 34:2666–2669. doi:10.1093/bioinformatics/bty149. 29547981 PMC6061794

[4] Kolmogorov M, Yuan J, Lin Y, Pevzner PA. 2019. Assembly of long, error-prone reads using repeat graphs. Nat Biotechnol 37:540–546. doi:10.1038/s41587-019-0072-8. 30936562

[5] Hunt M, Silva ND, Otto TD, Parkhill J, Keane JA, Harris SR. 2015. Circlator: automated circularization of genome assemblies using long sequencing reads. Genome Biol 16:294. doi:10.1186/s13059-015-0849-0. 26714481 PMC4699355

[6] Gurevich A, Saveliev V, Vyahhi N, Tesler G. 2013. QUAST: quality assessment tool for genome assemblies. Bioinformatics 29:1072–1075. doi:10.1093/bioinformatics/btt086. 23422339 PMC3624806

[7] Tatusova T, DiCuccio M, Badretdin A, Chetvernin V, Nawrocki EP, Zaslavsky L, Lomsadze A, Pruitt KD, Borodovsky M, Ostell J. 2016. NCBI Prokaryotic Genome Annotation Pipeline. Nucleic Acids Res 44:6614–6624. doi:10.1093/nar/gkw569. 27342282 PMC5001611

[8] Altschul SF, Gish W, Miller W, Myers EW, Lipman DJ. 1990. Basic local alignment search tool. J Mol Biol 215:403–410. doi:10.1016/S0022-2836(05)80360-2. 2231712

[9] Richter M, Rosselló-Móra R, Glöckner FO, Peplies J. 2015. JSpeciesWS: a Web server for prokaryotic species circumscription based on pairwise genome comparison. Bioinformatics 2015:929–931. doi:10.1093/bioinformatics/btv681. PMC5939971 26576653

